# Data on metagenomic profiles of bacterial endophyte communities associated with *Dicoma anomala*

**DOI:** 10.1016/j.dib.2022.108112

**Published:** 2022-03-28

**Authors:** Mehabo Penistacia Maela, Mahloro Hope Serepa-Dlamini

**Affiliations:** Department of Biotechnology and Food Technology, Faculty of Science, University of Johannesburg, Doornfontein Campus, P.O. Box 17011, Johannesburg 2028, South Africa

**Keywords:** Bacterial endophytes, *Dicoma anomala*, Bacterial endophyte diversity, 16S rRNA gene, Next generation sequencing, Illumina sequencing technology

## Abstract

Plants harbor varied communities of bacterial endophytes which play a crucial role in plant health and growth. *Dicoma anomala* is a medicinal plant that is known for its excellent ethnomedicinal uses which include treatment of coughs, fever, ulcers, and dysentery. This data in Brief article provides information on the diversity of bacterial endophytes associated with a medicinal plant, *Dicoma anomala* targeting the 16S rRNA gene using Illumina sequencing technology during three different seasons. Plant samples were collected in Eisleben, Limpopo province, South Africa, in the months of April, June, August and October 2018. The dataset revealed that the leaf samples collected in August had the highest species diversity as indicated by the Shannon index (4.25), Chao1 (1456.01), abundance-based coverage estimator (ACE) (1492.07) and the Simpson indices of diversity (0.05) irrespective of the species. The order of the bacterial endophyte's richness in *D. anomala* was April> October> June> August, from lowest to highest. The taxonomic composition analysis showed that most endophytic bacteria were composed of *Proteobacteria, Actinobacteria, Firmicutes, Bacteroidetes* and *Chloroflexi.* Some endophytic bacteria were found to be tissue specific. Sequences of *Cutibacterium, Acinetobacter* and *Methylobacterium* were prevalent in the leaves, whereas *Amycolatopsis* and *Bradyrhizobium* were the dominant genera in the root samples.

## Specifications Table

**Table:** 

Subject	Plant Science, Biology
Specific subject area	Molecular biology, Metagenomics, Bioinformatics
Type of data	Table Figure
How data were acquired	Metagenomic sequencing with of V3-V4 regions of the 16S rRNA genes using Illumina MiSeq at Agricultural Research Council (ARC), Onderstepoort, South Africa. Sequence processing of the metagenomic profiles was done using the EzBioCloud server (https://www.ezbiocloud.net/)
Data format	Raw Analysed Filtered
Parameters for data collection	Bacterial endophytes were isolated from fresh sterilized leaves and roots of a medicinal plant, *Dicoma anomala* obtained in Limpopo province, South Africa (23°31′50.2"S 29°48′46.7"E) during three seasons (autumn, winter, and spring).
Description of data collection	Metagenomic DNA extraction was performed using the modified method by Murray and Thompson [1]. The metagenomic profiles were sequenced with Illumina Miseq platform at Agricultural Research Council (ARC), Onderstepoort, South Africa. The sequence processing was done using the EzBioCloud server (https://www.ezbiocloud.net/). For characterization of community composition, the trimmed sequencing reads were de-noised using the PyroNoise algorithm and aligned against the customized SILVA database reference.
Data source location	Institution: University of Johannesburg City/Town/Region: Gauteng Country: South Africa Latitude and longitude (and GPS coordinates, if possible) for collected samples/data: 23°31′50.2"S 29°48′46.7"E
Data accessibility	Repository name: The Sequence Read Archive (SRA) of the National Centre for Biotechnology Information (NCBI) Data identification number: PRJNA576376 Direct URL to data: SAMN12993066, SAMN12993067, SAMN12993068, SAMN12993069, SAMN12993070, SAMN12993071, SAMN12993072 and SAMN12993073.

## Value of the Data

• This data will reveal the diversity of bacterial endophyte communities associated with a medicinal plant.
• The data will allow for comparison of bacterial endophyte communities between various plants.
• Knowledge about the bacterial endophyte communities will lead to extraction of information for further analysis on the plant-microbiome functions.

## 1 Data Description

Endophytes are non-pathogenic microorganisms that reside within the intracellular tissues of host plants without causing any harm. Endophytes are an essential component of the plant micro-bionetwork as they are known to play a vital role in plant growth, health, and productivity [2,3]. Metagenomics analyzes genetic material obtained directly from an environmental sample, along with other omics tools such as proteomics, transcriptomics and genomics has revolutionized the exploration of plant microbiota interactions by paving a way for culture independent methods through exploring microbial communities [4,5].

Metagenomic profiles of endophytic bacteria were isolated from surface sterilized leaves and roots of *Dicoma anomala*, targeting the 16S rRNA genes. Illumina sequencing technology was used to reveal the diversity of bacterial endophyte communities, define dominant taxa of bacterial endophytes from *Dicoma anomala* plant collected at different seasons, and to compare the bacterial endophyte communities hosted in this plant during different seasons in roots and leaves. *Dicoma anomala* is a medicinal plant that is distributed in Sub-Sahara Africa; in South Africa it is located in Gauteng, Limpopo, and Free-State provinces [6].

Following quality filtering, deletion of chimeras, singletons, mitochondrial and chloroplast sequences, a total of 214 060 reads were obtained from 7 samples. One sample was not included in the analysis of bacterial community structure due to a low number of sequence reads. The highest number of reads was obtained from the leaf tissues (85 316) collected in October, followed by the root tissues (60 204) collected in April. The leaves also had a lower number of reads (311) in April while for the roots the lowest number of reads was obtained in June with 579 reads as shown in Table 1. The sequences were assigned operational taxonomic units (OTU) clustering at a 97% cut-off similarity and a total of 3 675 OTUs were obtained after removing absolute singletons (Table 1). The OTUs were distributed among the 7 samples as indicated in Table 1. There were 1 863 OTUs in the overall spring dataset followed by 1 708 OTUs in the winter dataset and 104 OTUs in the autumn dataset, respectively. The roots samples had lower number of OTUs: 65 in autumn; 133 in winter and 817 in spring while for the leaves the lowest number of OTUs was observed in autumn with 39 OTUs. The highest number of OTUs observed for the leaves samples was in winter and spring with 1 575 and 1 046, respectively.

**Table 1:** Number of OTUs and *Alpha* diversity of bacterial endophytes in *D. anomala*

Sample name	Plant organ	Total reads	OTUs	ACE	CHAO	Shannon	Simpson
Met_DA1_H1	Roots	60204	65	137.18	133.3	0.69	0.56
Met_DA2_H2	Leaves	311	39	40.50	39.75	3.19	0.06
Met_DA3_H3	Roots	579	133	454.02	251.75	3.74	0.06
Met_DA4_H4	Leaves	2564	185	275.95	277.50	3.30	0.09
Met_DA5_CA1	Leaves	54196	1390	1492.07	1456.01	4.25	0.05
Met_DA7_reLO1	Leaves	85316	1046	1256.90	1163.68	2.72	0.25
Met_DA8_RO1	Roots	10890	817	1018.08	954.96	2.58	0.46

*Met_DA1_H1 and Met_DA2_H2 were collected in autumn (April): Met_DA3_H3 and Met_DA4_H4 in winter (June): Met_DA5_CA1 in winter (August): Met_DA7_reLO1 and Met_DA8_RO1 in spring (October).

Diversity indexes (Shannon and Simpson) between the roots and leaves indicated a significant difference between the roots and the leaves collected in the three seasons (p < 0.5). Although no significant differences were observed in the alpha diversity (Shannon index), we observed a significant difference of diversity of endophytic bacteria which was collected from the different months (seasons) (Table 1). However, grouping the diversity indices according to months (season), showed that the diversity of endophytic bacteria varied from one season to the other. The diversity index for the roots collected in autumn was significantly lower than in spring and winter while for the leaves the diversity index was significantly lower in autumn than in spring, early winter, and late winter (Table 1). Our observations showed the leaves sample from August (late winter) had the highest species diversity as shown by the Shannon index (4.25), Simpson index (0.05), Chao1 (1456.01) and ACE (1492.07) irrespective of the species. The order of the bacterial endophyte community richness in the samples was April> October > June> August, from lowest to highest.

The most dominant phyla in all the samples were *Proteobacteria, Actinobacteria, Firmicutes, Bacteroidetes* and *Chloroflexi*. *Proteobacteria* was the most abundant in all the 7 samples (42.83-99.95 %) followed by *Actinobacteria* (7.21-43.17%), Firmicutes (0.52-20.90 %) and *Bacteroidetes* (0.11-6.9 %). *Chloroflexi* was more abundant in leaves collected in August (2.89 %) and in the roots collected in October (2.61%) while in other samples it was found to be at a relative abundance of less than 1%.

**Fig. 1:**
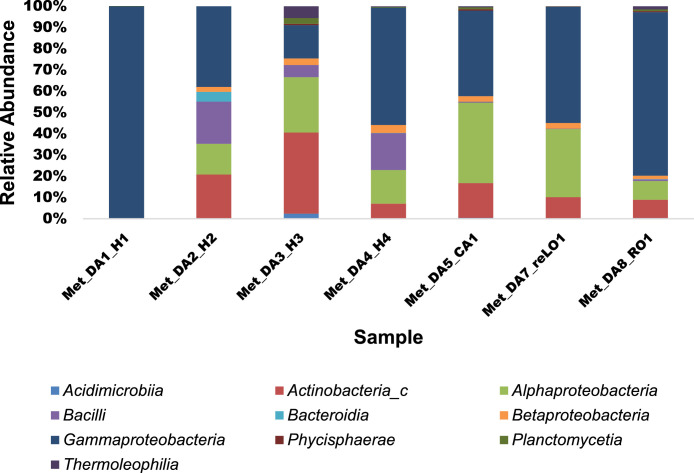
Taxonomic classification at the class level of bacterial endophytes in *Dicoma anomala*. The relative abundance (%) of the number of bacterial classes which were identified in *Dicoma anomala* among the seven samples.

At the class level (Fig. 1), two classes from *Proteobacteria* which were Gamma-proteobacteria (14.68-99.85%) and Alpha-proteobacteria (7.9-34.68%) were found to be the most abundant in all the samples. While *Actinobacteria* (7.72-35.75%) was the third dominating class in the phyla *Actinobacteria*. Within the phyla Firmicutes, *Bacilli* was only dominant in leaves collected in April (19.29%) and in both the roots (5.35%) and leaves (16.58%) collected in June, respectively. However, *Planctomycetia, Acidimicrobiia* and *Bacteroidia* were found at a relative abundance of less than 5%.

**Fig. 2:**
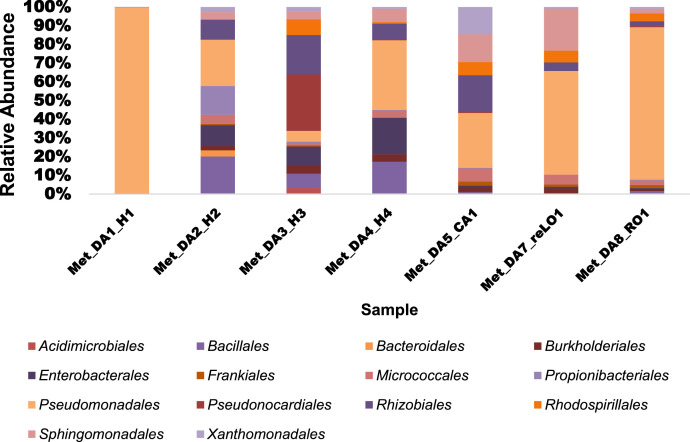
Taxonomic classification at the order level of bacterial endophytes in *Dicoma anomala*.

To assess the microbial composition, the most abundant phyla observed were *Proteobacteria, Actinobacteria* and *Firmicutes*. The Gamma-proteobacteria, had the highest number of Proteobacteria observed with the largest number of orders isolated (Fig. 2). The most important members detected included *Pseudomondales* (3.97-99.43%) and *Enterobacterales* (0.4-17.78%) in the class Gamma-proteobacteria. In the Alphaproteobacteria class, the most prominent members included *Rhizobiales* (2.0-16.26%) and *Sphingomonadales* (0.01-19.55%). In the Firmicutes class, *Bacillales* were found to be more abundant in the leaves (19.29%) collected in April and in the roots (5.35%) and leaves (15.76%) collected in June, whereas, in the remaining samples it was found to be at a relative abundance of less than 1%. The order *Pseudonocardiales,* was found to be more abundant in roots (20.29%) collected in June and it was not detected in the leaves collected in April.

The relative abundance (%) of number of bacterial orders identified in *Dicoma anomala* among the seven samples.

**Fig. 3:**
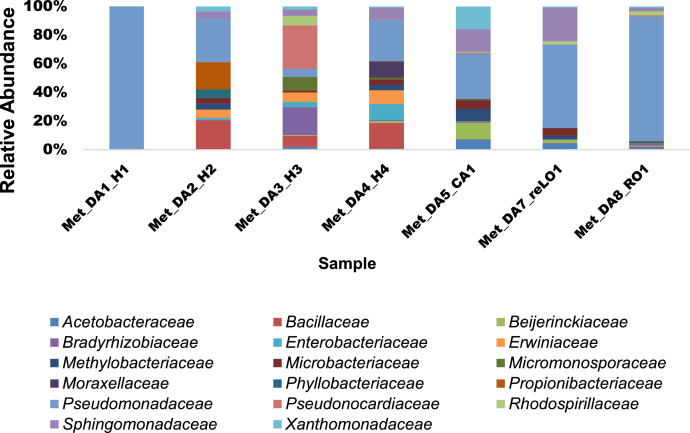
Taxonomic classification at the family level of bacterial endophytes in *Dicoma anomala*. The relative abundance (%) of the number of families identified in each sample.

A total of 18 families were identified. At the family level (Fig. 3), *Pseudomonadaceae* was the most abundant (3.9-99.44%) in all the samples, followed by *Sphingomonadaceae* (0.11-19.47%) and *Bacillaceae* (0.17-16.07%). However, *Pseudonocardiaceae* was more abundant in the roots (20.89%) collected in June whereas, *Propionibacteriaceae* was more abundant in the leaves (14.79) collected in April and found to be less than 1% in other samples.

**Fig. 4:**
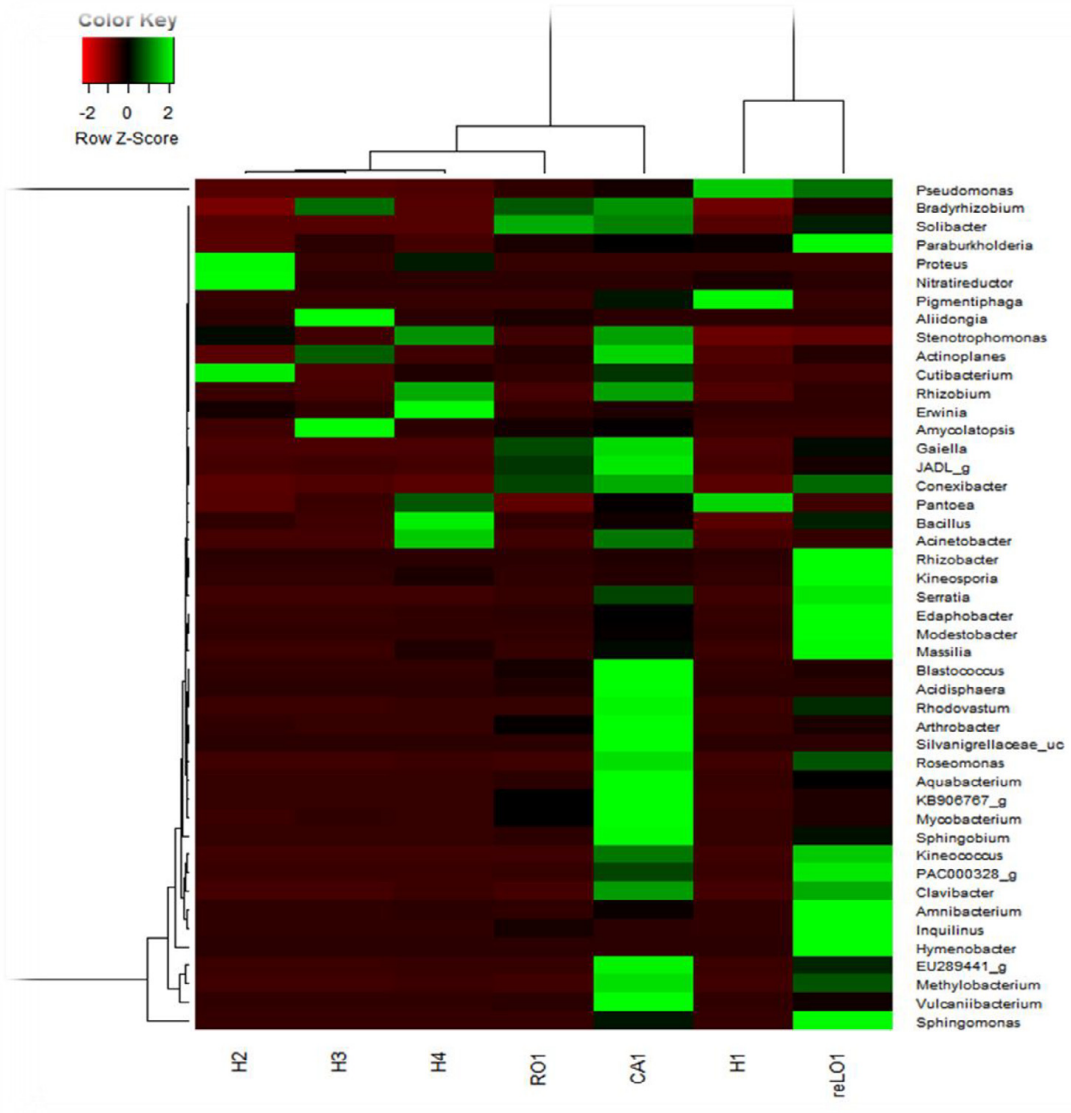
Taxonomic classification at the genus level of bacterial endophytes in *Dicoma anomala*. H1, H3 & RO1 are roots samples; H2, H4, CA1& reL01 are leaves samples.

At genus level (Table 2, and Fig. 4), the genera *Pseudomonas* (3.97-99.43%), *Sphingomonas* (0.01-18.77%) and *Bacillus* (0.01-16.07%) were the most dominant. In addition, the genera *Amycolatopis* (20.03%) and *Cutibacterium* (14.79%) were most dominant in the leaves collected in April whereas, *Bradyrhizobium* (11.97) was more dominant in the roots collected in June. Other genera such as *Pantoea* (0.00-5.57%), *Methylobacterium* (0.6.54%) and *Enterobacter* (0.912%) were also abundant genera with relative abundances greater than 5% in some samples. At the species level, *Pseudomonas fulva* species (2.76-68.69%) were the most dominant in all the samples followed by *Bacillus cereus* (0-13.10%) and *Pseudomonas_Uc* (0-12.86%).

**Table 2:** Taxonomic composition at the genus level in percentages.

Taxon Name	Met_DA1_H1	Met_DA7_reLO1	Met_DA8_RO1	Met_DA2_H2	Met_DA4_H4	Met_DA3_H3	Met_DA5_CA1
*Acinetobacter*	0	0,0141	0,0184	0	9,4774	0	0,3137
*Arthrobacter*	0	0,0399	0,5969	1,9293	0,039	0	0,7491
*Bacillus*	0,0017	0,1711	0,4408	16,0772	15,0156	5,3541	0,1587
*Bradyrhizobium*	0,005	0,0305	0,5693	0	0,429	11,9171	0,1476
*Caballeronia*	0	0,007	0,1194	0	0	0,1727	0,024
*Curtobacterium*	0	0,0035	0	0	0	0	0
*Enterobacter*	0	0,0082	0,0735	1,2862	0,195	0,3454	0,0111
*Enterobacteriaceae_g*	0	0,0094	0,0367	0	9,1264	1,8998	0,0295
*Hymenobacter*	0	6,4466	0	0	0,936	0	0,0092
*Methylobacterium*	0,01	2,1274	0,1286	3,2154	3,2371	0	6,5374
*Microbacteriaceae_uc*	0	0,0234	0,0551	0	0,039	0	0,9503
*Pseudomonas*	99,4353	48,3637	67,6951	23,7942	24,532	3,9724	24,011
*Pseudonocardia*	0	0,0047	0,0826	0	0	0,1727	0,2639
*Sphingomonas*	0,01	18,7761	1,0468	3,8585	6,3183	2,9361	7,8198
*Streptococcus*	0	0,0012	0,0092	0	0,468	0	0,0018
*Tepidisphaera*	0,0017	0,0492	0,3489	0	0,039	0,5181	0,5369
*Terrabacter*	0	0,0152	0,2479	0	0	0	0,214

*Met_DA1_H1 and Met_DA2_H2 were collected in autumn (April): Met_DA3_H3 and Met_DA4_H4 in winter (June): Met_DA5_CA1 in winter (August): Met_DA7_reLO1 and Met_DA8_RO1 in spring (October).

We observed that there are some endophytic bacteria that were plant tissue and season specific, whereas others were recovered from all the tissues. *Pseudomonas* was the most abundant genus in both the roots and the leaves of *D. anomala* (Figure 4). Members of the genus *Pseudomona*s provide important benefits to host plants by synthesizing phytohormones (IAA) and increasing host stress tolerance [7]. *Acinetobacter*, a common soil bacterium was more abundant in the leaves collected in June (winter), whereas *Acidibacter* was abundant in the roots collected in the same month/season; in the other seasons, these either absent or occurred at a relative abundance of less than 1%. *Bacillus* was more abundant in the leaves collected in April (autumn) and June (winter), while *Bradyrhizobium* was found to be abundant in the roots collected in June. Other endophytes were found to be organ specific. *Sphingobium* was not detected in the leaves of the first two months (April and June); it was only detected in August and was less abundant in October whereas, *Methylobacterium* was more prevalent in the leaves throughout all the seasons and occurred at a low abundance in the roots. Similar outcomes were observed for the genus, *Vulcaniibacterium.*

## 2 Experimental Design, Materials and Methods

### 2.1 Sample collection

The plant samples were collected from the same site but different plants in April, June, August, and October 2018 in Eisleben, Limpopo province, South Africa (23°31′50.2"S 29°48′46.7"E). Material collection of the plant included leaves and roots; with root collection, the plant was dug with precaution to minimize any damage. Immediately after collection, the plant was placed in sterile polyethylene bags and transported to the lab for further processing. To simplify and compare the differences of endophytic bacteria collected in different seasons, four groups were created as follows: 1) Met_DA1_H1 and Met_DA2_H2 collected in autumn (April), 2) Met_DA3_H3 and Met_DA4_H4 in winter (June), 3) Met_DA5_CA1 in winter (August) and 4) Met_DA7_reLO1 and Met_DA7_RO1 in spring (October).

### 2.2 Plant tissue sterilization

Plant roots and leaves were surface sterilized following the protocol by Hassan [8], with slight modifications. Briefly, the roots and the leaves were separately washed with running tap water to remove adhering soil particles, followed by a rinse with sterile distilled water prior to surface sterilization. The samples were sequentially washed by soaking in (i) 70% ethanol for 1 minute, (ii) 2. 5 % sodium hypochlorite for 5 minutes, (iii) 70 % ethanol for 30 sec, and (iv) rinsed five times in sterile distilled water to remove any traces of the solutions used. To confirm the success of sterilization, 100 µL of the last wash was plated on nutrient agar (NA) plates as control and incubated at 28°C for 24-72 hours. Effectiveness of the sterilization method was monitored on the control plates, with growth indicating poor sterilization. When growth had occurred, the plates were discarded, and the sterilization process was repeated. For the roots, the outer surfaces were trimmed out. The plant organs were then macerated in sterilized phosphate buffered saline (8 g NaCl, 0.2 g KCl, 1.44 g Na_2_HPO_4_, and KH_2_PO_4_ at pH 7.4). To ensure that epiphytes were removed, small parts of the roots and leaves were cut and plated on NA and incubated at 28 °C for 72 hours. Plates with no growth were selected for DNA extraction. Plant powders were stored at -22°C for future use.

### 2.3 Metagenomic DNA extraction

Total metagenomic DNA extraction was performed using the modified method described by Murray and Thompson [1]. Sterilized Eppendorf tubes were used to collect the powdered plant material and placed on ice. Briefly, a pre-heated solution of 2X Cetyltrimethylammonium bromide (CTAB) and 1 µL β-mercaptoethanol was added to the plant powders. The mixtures were vortexed for 20 seconds and incubated at 65°C for 1 hour. Following incubation, 600 µL chloroform/isoamyl (24:1 v/v) solution was added to each tube and inverted for 5 min. The tubes were centrifuged at 12 000 rpm for 5 min. The supernatant (∼500-550 µL) was collected and transferred to sterile Eppendorf tubes. An equal volume of ice-cold isopropanol and RNase (10 mg.ml^−1^ final concentration) was added to the supernatant and inverted. The tubes were incubated at room temperature for 20 min followed by centrifugation at 12 000 rpm for 5 min to recover the metagenomic DNA. The supernatant was discarded and pellets air dried. The DNA pellets were washed twice with 250 µL of 70% ethanol and centrifuged at 12 000 rpm for 5 min before drying in a laminar flow. The DNA was re-suspended in 50 µL nuclease free water and quantified using a Nanodrop ND-2000 UV-Vis spectrophotometer (Thermo Fisher Scientific, USA) before storage at -20°C for future use.

### 2.4 Amplicon metagenomic sequencing

The PCR library preparation involved steps to amplify the V3 and V4 regions using the 2X KAPA HiFi Hot Start Master Mix PCR kit and NextEra® XT index kit for attachment of dual indices and Illumina sequencing adapters. Hypervariable regions V3-V4 of the 16S rRNA gene were amplified using the forward primer:  5ʹ-CCTACGGGNGGCWGCAG-3ʹ and reverse primer 5ʹ-GACTACHVGGGTATCTA-3ʹ. The PCR reactions were performed with a 25 µL reaction containing 2.5 uL (5ng/uL) of genomic DNA, 5 uL of each primer (10 μM), 10.5 μL of 2 × KAPA HiFi HotStart MasterMix and 2μL of nuclease free water. The PCR reaction was carried out using the following conditions: initial denaturation at 95°C for 3 minutes followed by 25 cycles at 95°C for 30 s, 55°C for 30 s, and 72°C for 30 s and final extension at 72°C for 5 min. The PCR product was verified by running 1uL on a Bioanalyzer DNA 1000 chip. Nextera XT Index kit was used to attach dual indices and Illumina sequencing adapters, AMPure XP beads were used to clean PCR products pre- and post-tagging. The libraries were normalized and pooled prior to sequencing on Illumina MiSeq platform using MiSeq v3 reagent kit. The amplicon metagenomic sequencing was performed at the Biotechnology platform ARC, Onderstepoort, South Africa.

### 2.5 Sequence processing

The raw sequencing data of the seven samples were processed according to the Standard Operating Procedure (SOP) pipelines of the software package Mothur with slight modifications [9]. Briefly, the raw data of the reverse and forward reads were merged. The merged reads were filtered and trimmed by removing trailing bases with quality scored lower or equal to 2, maximum number of N was 4, maximum number of homopolymer was 8 and the contaminants were removed. For characterization of community composition, the trimmed sequencing reads were de-noised using the PyroNoise algorithm and aligned against the customized SILVA database reference [10]. The *de novo* Uchime algorithm was used to remove chimera, singletons, mitochondrial and chloroplasts sequences. Operational taxonomic units (OTUs) were build using the furthest neighbor clustering algorithm at a cut-off of 97% sequence similarity and classified using the naive Bayesian classifier which was trained against a customized Ribosomal Database Project (RDP) classifier training set, thus remaining only with bacterial-origin sequences [10,11]. Finally, the Alpha diversity indices were calculated as Shannon Index, implemented in Mothur.

### 2.6 Statistical analysis

Statistical analysis was performed using the One-way ANOVA to analyze all data obtained. Analysis was carried out using the Statistical Package for Social Science (SPSS) version 16.0 and the means were compared using Turkey's Studentized Range Test (HSD (0.05)) in the R program and p values < 0.05 were considered statistically different.

## CRediT Author Statement

**Mehabo Penistacia Maela:** Investigation, Software, Methodology, Data curation, Validation, Writing - original draft preparation; **Mahloro Hope Serepa-Dlamini:** Conceptualization, Supervisor, Writing – review & editing.

## Declaration of Competing Interest

The authors declare that they have no known competing financial interests or personal relationships that could have appeared to influence the work reported in this paper.

## Data Availability

Metagenome or environmental sample from plant metagenome (Original data) (Sequence Read Archive).
